# Development of a prognostic model for glioblastoma multiforme based on the expression levels of efferocytosis-related genes

**DOI:** 10.18632/aging.205422

**Published:** 2023-12-29

**Authors:** Wenzhe Xu, Lihui Han, Pengfei Zhu, Yufeng Cheng, Xuan Chen

**Affiliations:** 1 Department of Neurosurgery, Qilu Hospital of Shandong University and Institute of Brain and Brain-Inspired Science, Shandong University, Shandong, Jinan 250012, China; 2 Department of Radiation Oncology, Qilu Hospital of Shandong University, Shandong, Jinan 250012, China

**Keywords:** glioblastoma multiforme, efferocytosis, prognostic model, immune microenvironment, macrophages

## Abstract

Glioblastoma multiforme (GBM) is one of the most common and aggressive brain tumors. The microenvironment of GBM is characterized by its highly immunosuppressive nature with infiltration of immunosuppressive cells and the expression levels of cytokines. Efferocytosis is a biological process in which phagocytes remove apoptotic cells and vesicles from tissues. Efferocytosis plays a noticeable function in the formation of immunosuppressive environment. This study aimed to develop an efferocytosis-related prognostic model for GBM. The bioinformatic methods were utilized to analyze the transcriptomic data of GBM and normal samples. Clinical and RNA-seq data were sourced from TCGA database comprising 167 tumor samples and 5 normal samples, and 167 tumor samples for which survival information was available. Transcriptomic data of 1034 normal samples were collected from the Genotype-Tissue Expression (GTEx) database as a control sample supplement to the TCGA database. In the end, 167 tumor samples and 1039 normal samples were obtained for transcriptome analysis. Efferocytosis-related differentially expressed genes (ERDEGs) were obtained by intersecting 7487 differentially expressed genes (DEGs) between GBM and normal samples along with 1189 hub genes. Functional enrichment analyses revealed that ERDEGs were mainly involved in cytokine-mediated immune responses. Moreover, 9 prognosis-related genes (PRGs) were identified by the least absolute shrinkage and selection operator (LASSO) regression analysis, and a prognostic model was therefore developed. The nomogram combining age and risk score could effectively predict GBM patients’ prognosis. GBM patients in the high-risk group had higher immune infiltration, invasion, epithelial-mesenchymal transition, angiogenesis scores and poorer tumor purity. In addition, the high-risk group exhibited higher half maximal inhibitory concentration (IC50) values for temozolomide, carmustine, and vincristine. Expression analysis indicated that PRGs were overexpressed in GBM cells. PDIA4 knockdown reduced efferocytosis *in vitro*. In summary, the proposed prognostic model for GBM based on efferocytosis-related genes exhibited a robust performance.

## INTRODUCTION

Glioblastoma multiforme (GBM) stands as the most prevalent malignant type of primary brain tumors in adults, accompanying by a poor prognosis and a high mortality rate [1]. It accounts for approximately 14.2% of all brain tumors and 50.1% of all malignant tumors according to the most recent statistics presented by the Central Brain Tumor Registry of the United States (CBTRUS) [2]. The current first-line treatments for GBM include maximal surgical tumor resection, followed by radiotherapy plus concurrent and adjuvant temozolomide (TMZ) chemotherapy. However, recurrence seems to be the rule [3]. Despite the integration of tumor-treating fields (TTFs), the median overall survival for GBM only experiences a modest extension from 16.0 to 21.6 months, which is primarily attributable to the tumor’s aggressive nature and resistance to conventional treatments [4, 5]. In recent years, molecular-targeted therapies and immunotherapy have been widely utilized in clinical trials [6]. However, patients’ clinical outcomes exhibit noticeable diversity, a reflection of the pronounced molecular and cellular heterogeneity inherent in GBM. Biomarkers are used to quantify normal physiology and physiological response to therapies. Due to the challenges of these treatments identification of new predictive and prognostic biomarkers to gauge response to immune therapy for patients with GBM will be critical in the precise treatment of this highly heterogenous disease [7]. Therefore, it is essential to find new efficient diagnostic biomarkers and therapeutic targets for GBM.

Throughout the progression of GBM, tumor cell apoptosis is widespread, and this phenomenon intensifies with the application of cytotoxic treatments, such as chemotherapy and radiotherapy. Apoptotic tumor cells are rapidly and efficiently detected and cleared by professional and non-professional phagocytes through efferocytosis, preventing secondary necrosis [8]. Equally important, engulfing phagocytes may release anti-inflammatory substances and recruit myeloid-derived suppressor cells (MDSCs) and regulatory T (Treg) cells to enforce immune tolerance [9]. Efferocytosis plays a pivotal role in the maintenance of tissue homeostasis in normal physiology and the restoration of homeostasis during inflammation. In contrast, tumor cells can exploit the immunosuppressive environment facilitated by efferocytosis to elude immune surveillance and advance tumor progression [10]. The microenvironment of GBM is characterized by its highly immunosuppressive nature with infiltration of immunosuppressive cells and the expression levels of cytokines. Microglia/macrophages, as the major professional phagocytes of the central nervous system, are more abundant in GBM compared with low-grade gliomas [11]. Efferocytosis has the potential to prompt the polarization of tumor-associated macrophages, steering them towards an anti-inflammatory M2 phenotype, promoting pro-tumor activation [12]. Tyrosine kinase Mer (MerTK), the main receptor of efferocytosis, is overexpressed in GBM cells, and it is associated with invasion and survival of GBM cells [13]. Regarding the enriched apoptosis and immune-suppressive microenvironment in GBM, the role of efferocytosis-related genes in GBM is worthy of further investigation.

In the present study, bioinformatics methods, such as differential expression analysis and weighted gene co-expression network analysis (WGCNA), were employed to develop a novel efferocytosis-related prognostic gene signature for GBM. Novel efferocytosis-related prognostic gene signature has the potential to be used in clinical practice for risk stratification of GBM patients and for selecting individuals who are likely to respond to immunotherapy. This can help clinicians design appropriate targeted therapies before initiating clinical treatment [14] In addition, in-depth analyses encompassing signal transduction pathways, the tumor microenvironment, and responsiveness to anticancer drugs hold promise for revealing novel insights into the molecular mechanisms underlying GBM.

## RESULTS

### Efferocytosis-related differentially expressed genes (ERDEGs) were mainly involved in cytokine-mediated immune responses

**Figure 1:**
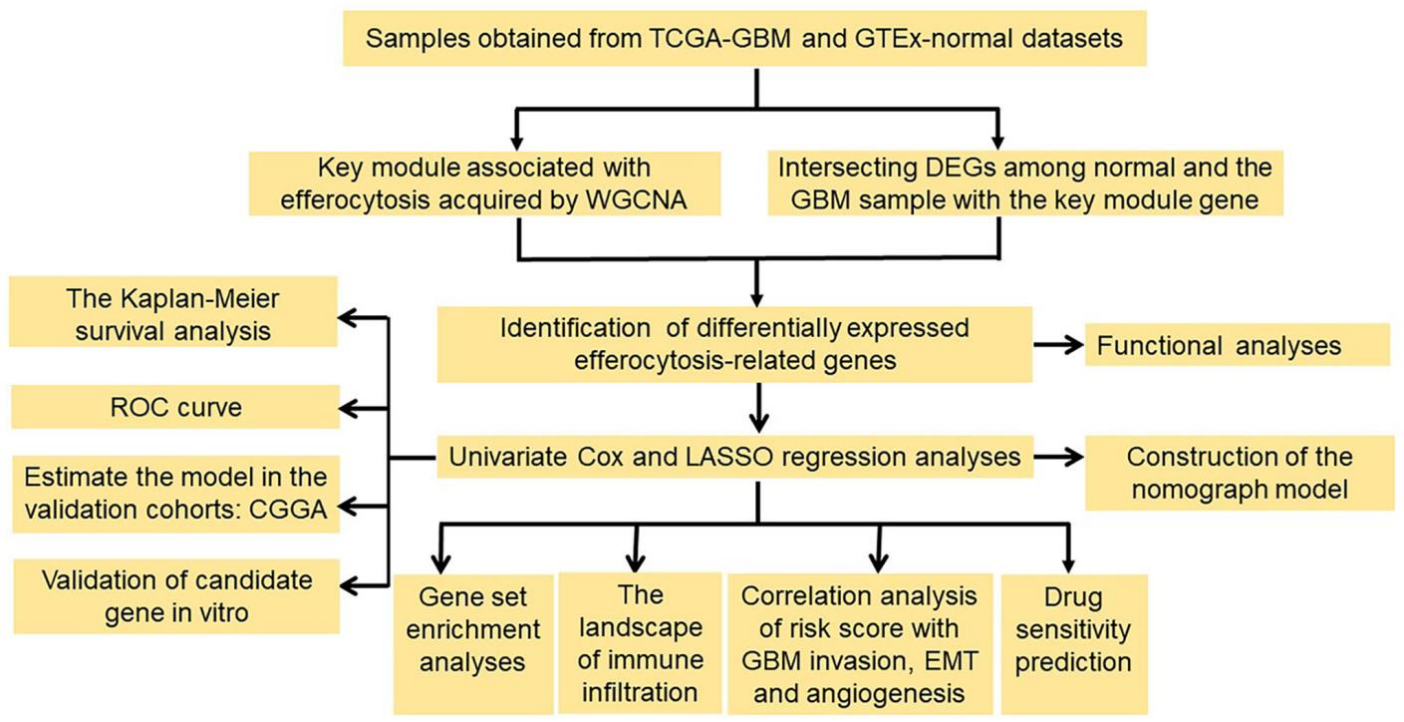
**Flowchart of the study.** Abbreviations: WGCNA: weighted gene co-expression network analysis; DEGs: differentially expressed genes; ROC: the receiver operating characteristic; LASSO: the least absolute shrinkage and selection operator.

**Figure 2:**
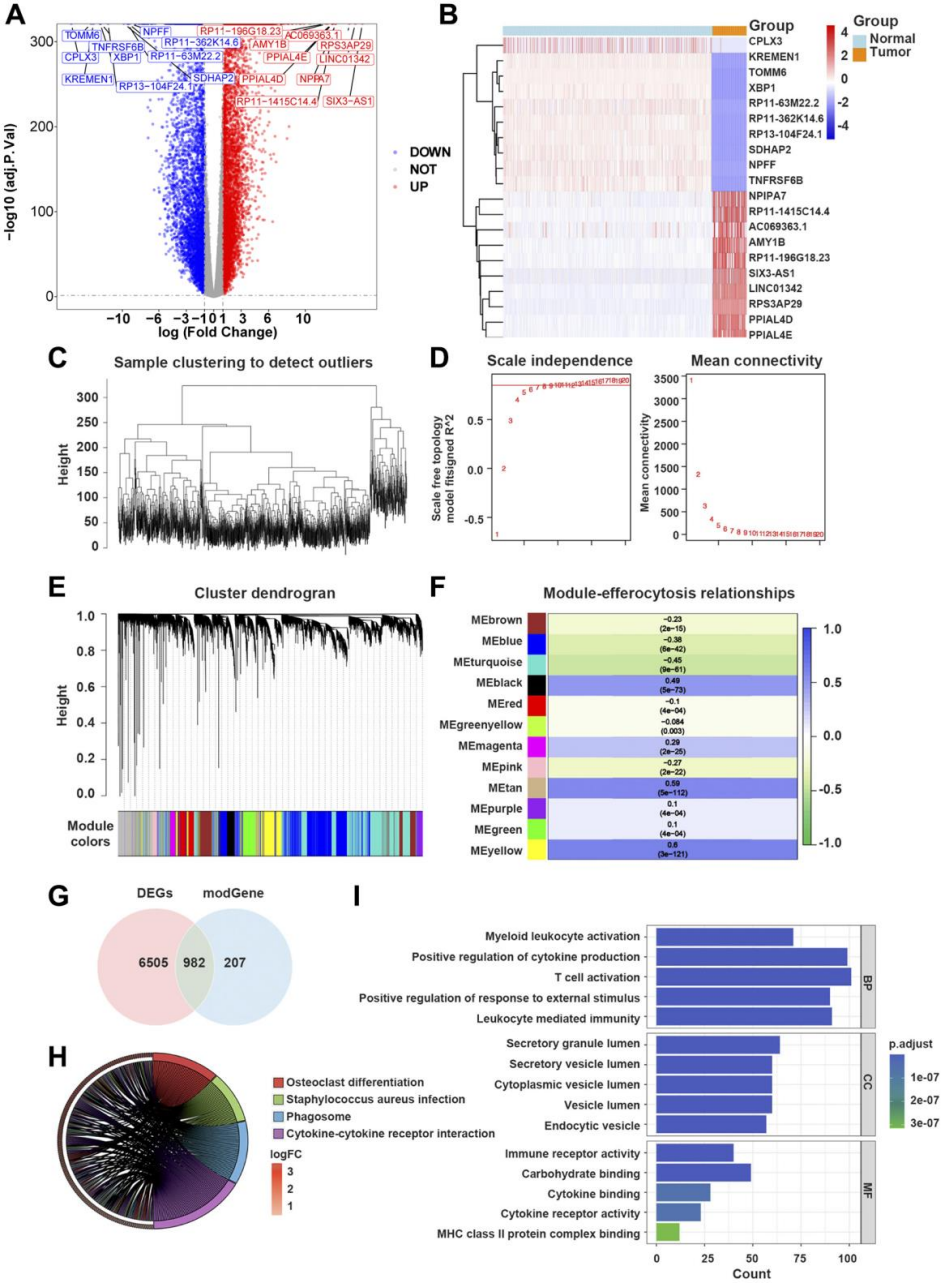
**Efferocytosis-related differentially expressed genes (ERDEGs) were primarily involved in immune responses mediated by cytokines.** (**A**) Volcano plot of DEGs in glioblastoma multiforme (GBM). (**B**) Heat map of top 10 upregulated and top 10 downregulated genes in GBM. (**C**) The Hclust function indicated that all samples in the training dataset were well clustered. (**D**) The soft-threshold power of 10 was selected to calculate adjacencies based on R2 > 0.85. (**E**) 12 modules were identified by dynamic tree cutting approach. (**F**) MEyellow and MEtan, which were mainly related to efferocytosis scores were selected, and 1,189 genes were considered as hub genes. (**G**) 982 ERDEGs were identified by crossing 1,189 hub gens with 7,487 DEGs found between GBM and normal samples. (**H**) Kyoto Encyclopedia of Genes and Genomes (KEGG) pathway analysis of ERDEGs. (**I**) Gene Ontology (GO) enrichment analysis of ERDEGs.

The study flowchart is illustrated in Figure 1. Through differential analysis, 7487 differentially expressed genes (DEGs) in normal and GBM samples were screened, comprising 4374 downregulated DEGs and 3113 upregulated DEGs (Figure 2A, 2B). All samples in the training dataset exhibited cohesive clustering, devoid of any outliers (Figure 2C). In order to construct a co-expression network and distinguish modules, the WCGNA was performed. First, the soft-threshold power of 10 was selected to calculate adjacencies (Figure 2D). Subsequently, 12 modules were obtained by the dynamic tree cutting approach (Figure 2E). Among the modules, MEyellow and MEtan, which were highly associated with efferocytosis scores, were selected (|cor| > 0.5 and *P* < 0.05). The MEyellow and the MEtan contained 1,189 genes that were used as hub genes (Figure 2F). Furthermore, 982 ERDEGs were obtained by crossing 1,189 hub genes and 7487 DEGs between the GBM and normal samples (Figure 2G).

The outcomes of the Kyoto Encyclopedia of Genes and Genomes (KEGG) pathway analysis revealed that ERDEGs were primarily associated with the differentiation of osteoblasts, *Staphylococcus aureus* infection, phagosomes, cytokine-cytokine receptor interaction, etc., (Figure 2H).

The Gene Ontology (GO) analysis suggested that ERDEGs were remarkably abundant in the activation of myeloid leukocytes, cytokine-mediated positive regulation, activation of T cells, leukocyte-mediated immunity, activity of immune receptors, binding of cytokines, etc., (Figure 2I).

### The ERDEGs-based prognostic model could accurately assess the prognosis of GBM patients

**Figure 3:**
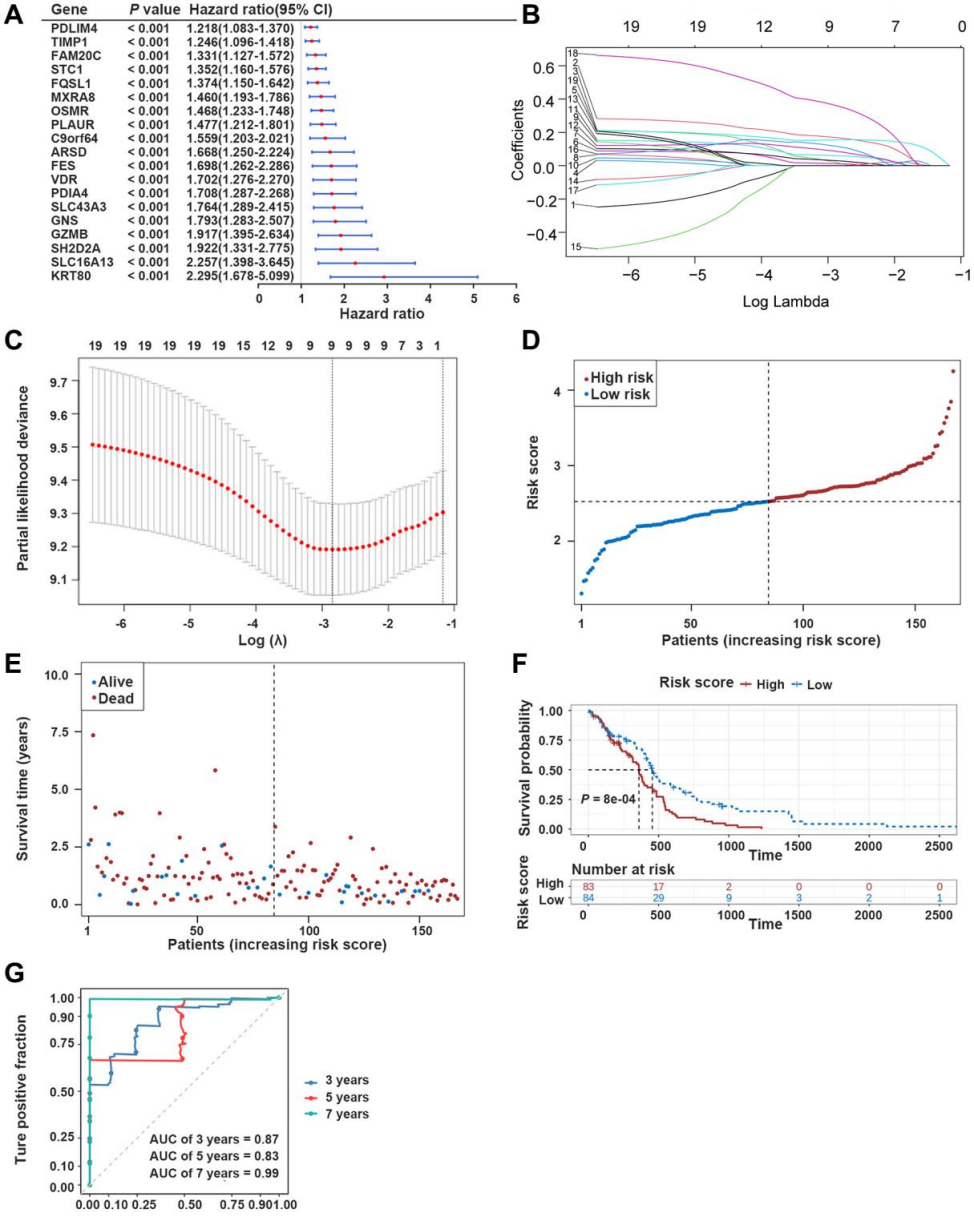
**Development of the prognostic model based on ERDEGs.** (**A**) Univariate Cox analysis filtered out 19 ERDEGs correlated with GBM patients’ survival. (**B**, **C**) LASSO regression analysis revealed that 9 ERDEGs were prognosis-related genes (PRGs) of GBM patients. (**D**) Distribution of patients based on the risk score in the training dataset. (**E**) Survival time and status of patients in the training dataset. (**F**, **G**) The Kaplan-Meier (K-M) survival curve and ROC curve in the training dataset.

In The Cancer Genome Atlas (TCGA)-GBM dataset, the univariate Cox analysis of ERDEGs was conducted to screen 19 survival-associated ERDEGs in GBM patients (HR≠1, *P* < 0.001) (Figure 3A). Furthermore, the results of the least absolute shrinkage and selection operator (LASSO) regression analysis indicated that the optimal model was developed when lambda was 0.058, and the 9 ERDEGs (PDIA4, GNS, OSMR, MXRA8, PDLIM4, STC1, C9orf64, SLC16A13, and GZMB) were prognosis-related genes (PRGs) of GBM patients (Figure 3B, 3C). The distribution of patient risk scores and survival status is shown in Figure 3D, 3E. The survival analysis indicated that the prognosis of high-risk group patients with the relatively short survival time accounted for the majority of GBM patients. Subsequently, the Kaplan-Meier (K-M) survival curves revealed that compared with the low-risk group, patients in the high-risk group significantly exhibited shorter survival time (*P* < 0.05) (Figure 3F). In the training dataset, risk score was found to be able to reliably assess GBM (Figure 3G).

**Figure 4:**
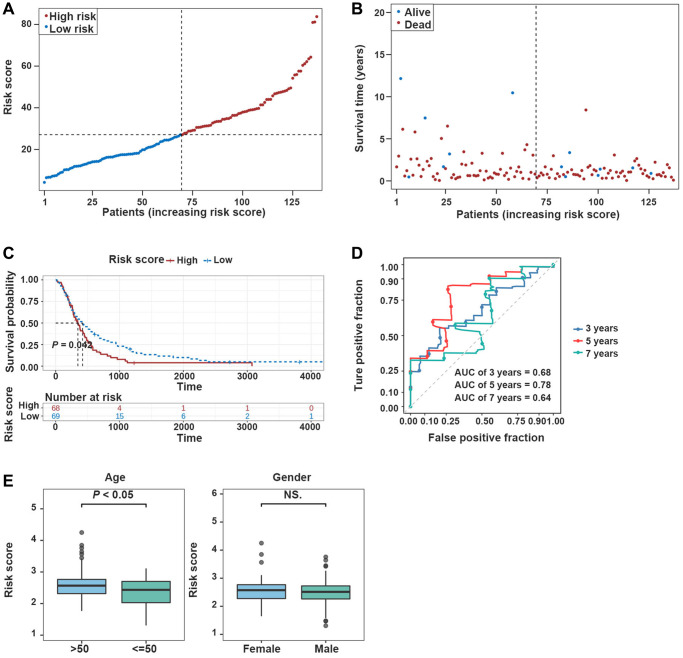
**Validation of the prognostic model in the validation dataset.** (**A**, **B**) Risk plot distribution in the validation dataset. (**C**, **D**) The K-M survival curve and the ROC curve in the validation dataset. (**E**) Box plot of correlation between risk score and clinical features (Age and Gender).

Afterward, the prediction ability of the prognostic model was confirmed using the validation dataset. The high-risk group was enriched with GBM patients whose median survival time was relatively short (Figure 4A, 4B). The K-M survival curves revealed significantly poorer survival in the high-risk group compared with the low-risk group (Figure 4C). In the validation dataset, the risk score proved to be reliable in assessing GBM (Figure 4D).

Additionally, risk scores were higher in TCGA-GBM dataset for patients who aged >50 years (*P* < 0.05), and risk scores were independent of patients’ gender (Figure 4E).

### The nomogram model could reliably predict GBM patients’ prognosis

**Figure 5:**
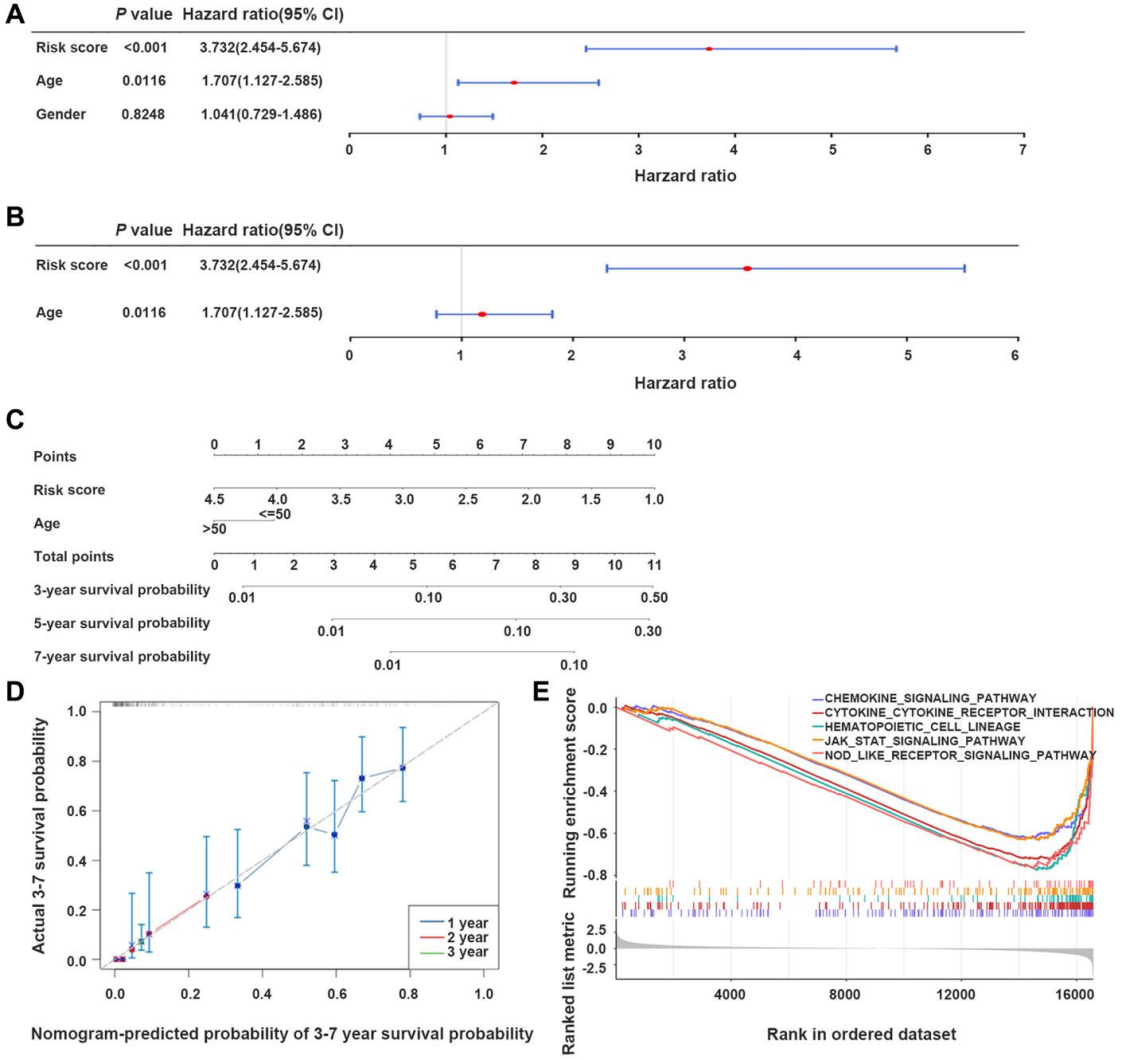
**The nomogram model developed for predicting GBM patients’ prognosis.** (**A**, **B**) Univariate and multivariate Cox regression analyses of the integration of risk scores and clinical parameters. (**C**) Development of a prognostic nomogram model for estimating GBM patients’ prognosis. (**D**) Calibration curve of the nomogram model at 3-, 5-, and 7-year. (**E**) Gene set enrichment analysis (GSEA) results, indicating the cytokine-cytokine receptor interaction, and JAK-STAT signaling pathway.

Univariate Cox analysis confirmed the correlation between risk score and age with GBM patients’ survival in TCGA-GBM dataset (*P* < 0.05) (Figure 5A). Next, the independent prognostic value of risk score was identified by multivariate Cox regression analysis (*P* < 0.05) (Figure 5B).

The integration of the risk score with age promoted the development of a nomogram model, providing a reliable tool to estimate GBM patients’ prognosis (Figure 5C, 5D).

### Genes in the high- and low-risk groups were associated with the JAK-STAT-mediated signaling pathway

In TCGA-GBM dataset, gene set enrichment analysis (GSEA) demonstrated that genes in the two groups primarily participated in signaling pathways, such as chemokines, cytokine-cytokine receptor interaction, and the JAK-STAT signaling pathway (Figure 5E).

### The high-risk score was characterized by the elevated immune infiltration and poor tumor purity

**Figure 6:**
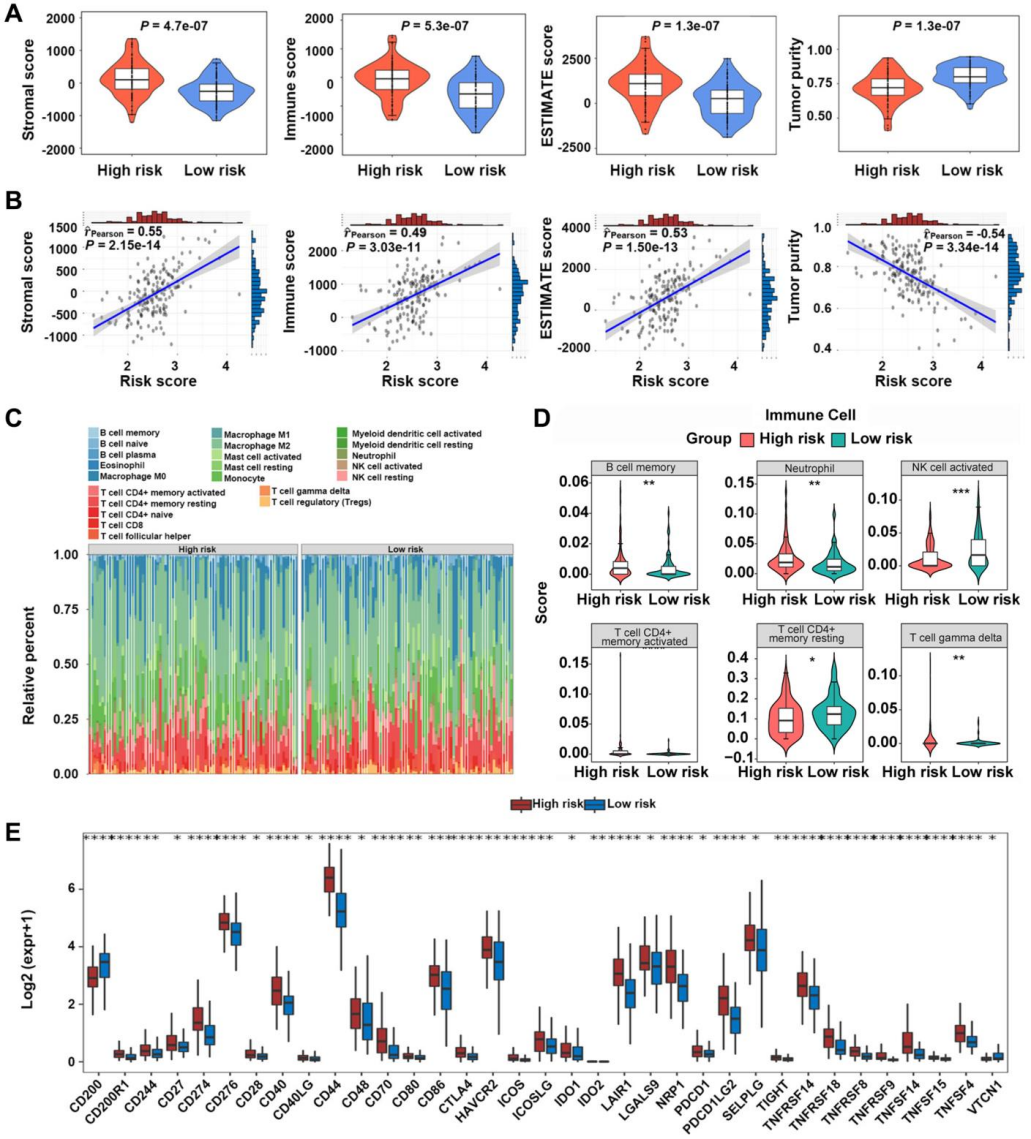
**Immune microenvironment analysis between high-risk and low-risk groups.** (**A**, **B**) Relationship between risk scores and GBM microenvironment scores. (**C**, **D**) Comparison of the infiltration levels of 22 immune cells in high-risk and low-risk groups. (**E**) Comparison of the expression levels of immune checkpoints between the two groups. ^*^P < 0.05, ^**^P < 0.01, ^***^P < 0.001.

In TCGA-GBM dataset, it was noted that samples from the high-risk group exhibited significantly higher stromal, ESTIMATE, and immune scores compared with those from the low-risk group (*P* < 0.05) (Figure 6A). Tumor purity was significantly less in the high-risk group compared with that in the low-risk group (*P* < 0.05) (Figure 6A). It was revealed that the risk scores of GBM patients were positively correlated with stromal, ESTIMATE, and immune scores, and they were negatively associated with tumor purity (Figure 6B). Furthermore, the infiltration levels of B cell memory, neutrophil, and resting T cell gamma delta were significantly different between the two groups (Figure 6C, 6D). The expression levels of 35 immune checkpoints were significantly different between the two groups (Figure 6E).

### The high-risk score was characterized by the elevated invasion, epithelial-mesenchymal transition (EMT), and angiogenesis scores

**Figure 7:**
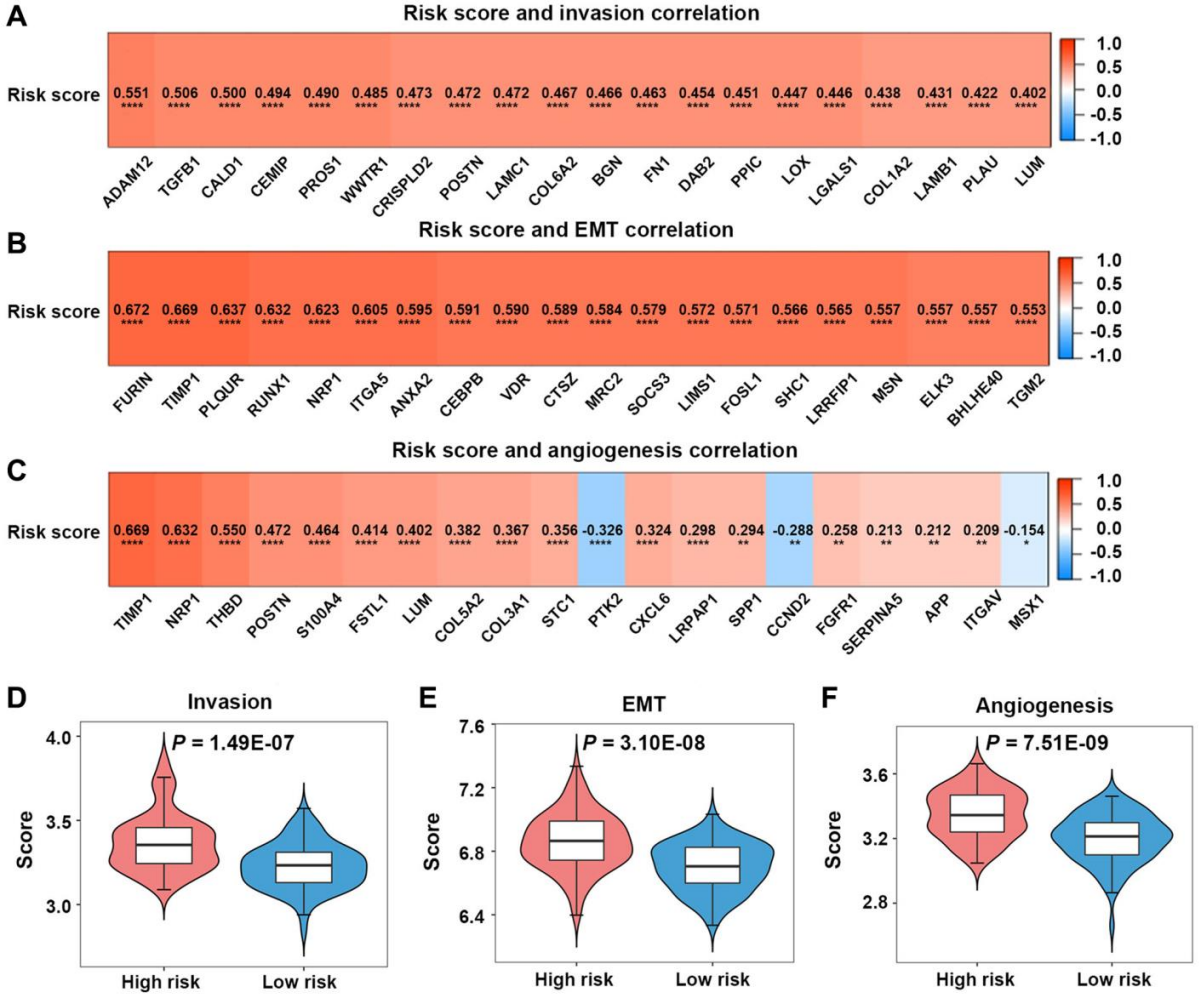
**Invasion, epithelial-mesenchymal transition (EMT), and angiogenesis analyses in the high-risk and low-risk groups.** (**A**–**C**) Relationships between risk scores and invasion-, EMT-, angiogenesis-related genes. (**D**–**F**) Comparison of invasion, EMT, and angiogenesis scores between the two groups.

In TCGA-GBM dataset, the invasion-associated genes (ADAM12, TGFBI, CALD1, and CEMIPPROS1) were found to be related to the risk score (Figure 7A). The EMT-associated genes (FURIN, TIMP1, PLAUR, RUNX1, and NRP1) were noted to be related to the risk score (Figure 7B). The angiogenesis-associated genes (TIMP1, NRP1, THBD, POSTN, and S100A4) were found to be related to the risk score (Figure 7C).

Patients in the high-risk group in TCGA-GBM dataset had significantly greater invasion, EMT, and angiogenesis scores than those in the low-risk group (*P* < 0.05) (Figure 7D–7F).

### Correlation analysis of the prognostic model and the sensitivity of chemotherapeutic agents

**Figure 8:**
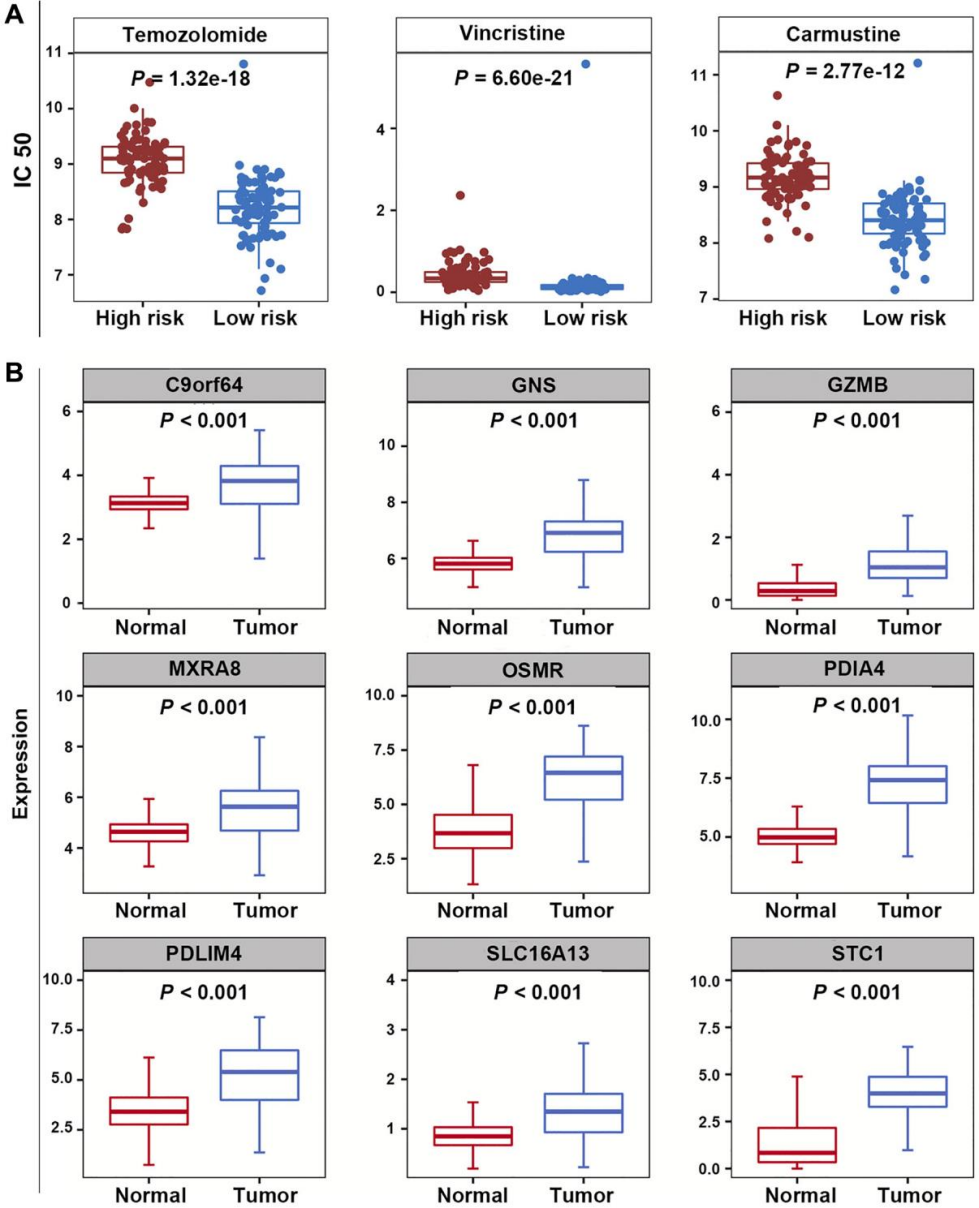
**Correlation analysis of prognostic model and chemotherapeutic sensitivity, as well as expression analysis of PRGs.** (**A**) The IC50 values for temozolomide, carmustine, and vincristine in the high-risk and low-risk groups. (**B**) The expression levels of nine PRGs.

The half maximal inhibitory concentrations (IC50) for TMZ, carmustine, and vincristine were significantly lower in the low-risk group, suggesting that the efferocytosis genes-based prognostic model could predict the potential value of chemotherapy for GBM patients (*P* < 0.05) (Figure 8A).

### PRGs were highly expressed in GBM samples

In the training dataset, compared with normal samples, the expression levels of 9 PRGs were significantly elevated in GBM samples (*P* < 0.05) (Figure 8B).

### PDIA4 knockdown in GBM cells reduced efferocytosis *in vitro*

**Figure 9:**
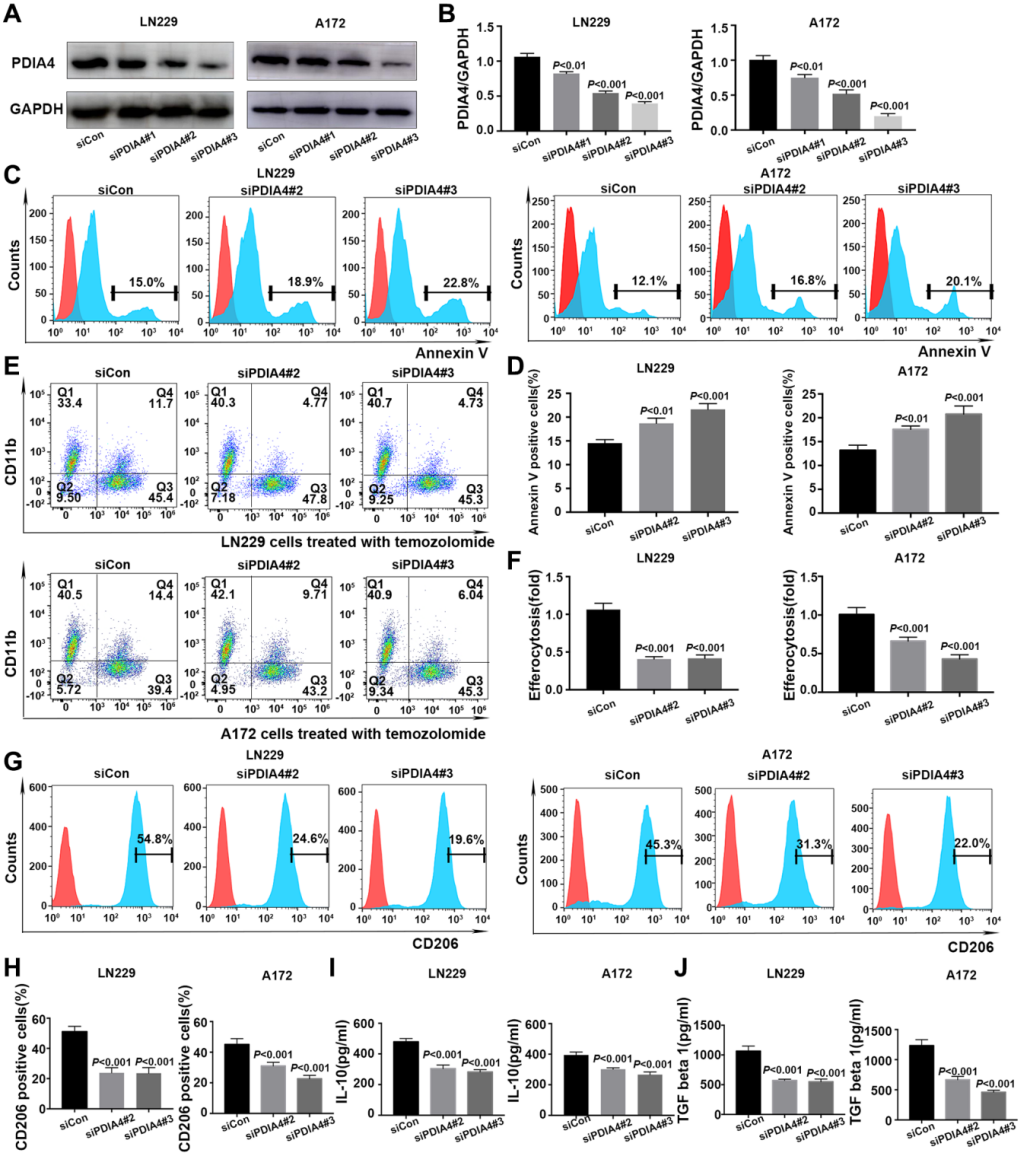
***In vitro* validation of PDIA4 in GBM efferocytosis.** (**A**, **B**) PDIA4 knockdown efficacy was detected by Western blotting. (**C**, **D**) Exposure of phosphatidylserine on apoptotic cell surface was evaluated by Annexin V level. (**E**, **F**) Efferocytosis was indicated by proportion of CD11b^+^CFSE^+^ cells. (**G**, **H**) Quantification of the expression level of macrophages-associated marker (CD206) by flow cytometry. (**I**, **J**) Measurement of IL-10 and TGF-β levels in cocultured medium of efferocytosis assays. Data are presented as mean ± SD.

PDIA4 was selected to further validate the performance of efferocytosis-related prognostic model. After confirming the knockdown effect of siRNA-PDIA4 by detecting the expression level of Erp72 (a protein product of PDIA4) (Figure 9A, 9B), TMZ was utilized for inducing apoptosis of LN229 cells in different groups (Figure 9C). Notably, the exposure of phosphatidylserine (PS) on the apoptotic cell surface was enhanced upon the knockdown of PDIA4 (Figure 9D). However, the inhibition of PDIA4 hindered efferocytosis in GBM cells and the THP-1-derived macrophages coculture system (Figure 9E, 9F). The expression levels of M2 macrophages-expressed marker CD206 (Figure 9G, 9H) and secreted factors interleukin-10 (IL-10) (Figure 9I) and transforming growth factor-β (TGF-β) (Figure 9J) were also reduced in PDIA4 knockdown group.

## DISCUSSION

Despite the utilization of multi-therapeutic strategies, GBM patients’ prognosis remains poor due to its high aggressiveness nature. Patient outcomes in GBM exhibited a remarkable variability due to the pronounced molecular and cellular heterogeneity of the disease. It is clinically valuable to explore novel prognostic markers and therapeutic targets for GBM. Tumor cells adeptly exploit the immunosuppressive microenvironment established by efferocytosis to evade immune surveillance and foster tumor progression [10]. However, few studies have concentrated on the relationship between efferocytosis and GBM.

In the present study, a novel efferocytosis-related prognostic gene signature for GBM was first developed and validated. Efferocytosis maintains tissue homeostasis and supports the resolution of inflammation and injury by engulfing apoptotic cells and producing anti-inflammatory substances [15]. Macrophages and microglia are the major professional phagocytes, accounting for the vast majority of dominant infiltrating immune cells in the GBM microenvironment [16]. Once the apoptotic cells are engulfed by phagocytes, phagosomes can be modified to influence the degradation of the cell corpse following fusion with lysosome [17]. The ERDEGs were primarily associated with phagosomes according to the results of the KEGG pathway analysis. The anti-inflammatory cytokines released after efferocytosis could recruit MDSCs and Treg cells, and regulate the polarization of macrophages form the M1 phenotype to the M2 phenotype for reprogramming an immunosuppressive tumor microenvironment [9, 12]. The ERDEGs were significantly abundant in the myeloid leucocyte activation, T cell activation, and cytokine-cytokine receptor interaction according to the results of GO enrichment analysis.

Using LASSO regression analysis, 9 PRGs (OSMR, STC1, PDIA4, MXRA8, PDLIM4, C9orf64, and GZMB) were identified in GBM patients. The majority of the genes were found to be associated with different biological mechanisms of GBM, except for GNS and SLC16A13. The cytokine receptor for oncostatin M (OSMR) and stanniocalcin-1 (STC1) have exhibited to reprogram the immune microenvironment [18, 19]. OSM, an IL-6 family cytokine in inducing mesenchymal properties in GBM which mediated signaling contributes to aggressive nature associated with mesenchymal features via STAT3 signaling in glioma cells [20]). The mesenchymal-like state of glioblastoma, which is associated with the increased cytotoxicity of T cells, was induced by macrophages-derived OSM interacting with OSMR [18]. STC1 is a hormone-like glycoprotein that has shown to regulate homeostasis of calcium and phosphorus. The tumor’s secreted STC1 has been identified to interact with calreticulin (CRT), an “eat-me” signal, leading to reduced CRT membrane exposure. This reduction acts as a deterrent against phagocytosis by dendritic cells and macrophages [19]. STC1 was found to be highly expressed in GBM cells, enhancing the stem-like features of glioblastoma cells [21]. In addition, STC1 regulates GBM migration and invasion via the TGF-β/SMAD4 signaling pathway [22]. Previous studies have reported the involvement of PDIA4, MXRA8, PDLIM4, C9orf64, and GZMB in glioma patients’ poor prognosis through bioinformatics analysis. Protein disulfide isomerase A4 (PDIA4) is a member of the multi-protein chaperone complex, performing diverse functions by interacting with its specific substrates [23]. A growing body of evidence demonstrated that aberrant PDIA4 expression level and its potential mechanisms participate in the development of numerous types of cancer, including GBM [24]. PDIA4 is a key promotor of GBM. PDIA4 regulates the proliferation via activating the PI3K/AKT/m-TOR pathway and suppression of apoptosis in GBM [25]. By interacting with a variety of immunological components, PDIA4 cold promote an immunosuppressive microenvironment in tumor [26]. Matrix remodeling-associated protein 8 (MXRA8) was found to be overexpressed in glioma cells, and it could be involved in the infiltration of M2 macrophages, contributing to immune response to glioma by regulating ferroptosis [27]. PDLIM4, an actin-binding protein containing PDZ and LIM domains, has been implicated as a tumor suppressor in prostate cancer and ovarian cancer due to its hypermethylation or downregulation feature [28]. However, it could be significantly upregulated in GBM cells along with other genes in high-grade gliomas, suggesting that PDLIM4 may have a potential oncogenic function [29, 30]. GZMB transcription is regulated by nuclear factor of activated T cells, Ikaros, and AP-1. GZMB gene transcription is also activated and enhanced by NF-κB and by Janus kinase 1/signal transducer and activator of transcription signaling. The role of GZMB in apoptosis makes it an attractive anticancer target [31]. C9orf64 and GZMB were identified as the prognostic biomarkers for GBM by bioinformatics analysis [32, 33]. In the present study, the expression levels of above-mentioned genes were significantly elevated and associated with GBM patients’ survival.

Glucosamine (N-Acetyl)-6-sulfatase encoded by GNS is a lysosomal enzyme that is involved in the catabolism of heparan sulfate and glycosaminoglycans [34]. GNS-deficient mice exhibited widespread neuroinflammation, as evidenced by the activation of microglial cells [35]. The expression level of GNS was found to be correlated with the magnitude of immune response [36]. Recent research demonstrated that the solute carrier (SLC) family of membrane transport proteins plays a role in modulating efferocytosis by upregulating the expression levels of anti-inflammatory genes and sustaining continuous efferocytosis [37]. It was reported that SLC16A13 could be a potential biomarker for tumor prognosis, including oral squamous cell carcinoma, lung adenocarcinoma, and pancreatic cancer [38–40]. However, the relationship between these two genes and GBM has not yet been explored.

To further explore the potential molecular mechanisms of these efferocytosis-related genes, GSEA was conducted and it was demonstrated that they mainly participated in the signaling pathway of chemokines, cytokine-cytokine receptor interaction, and JAK-STAT signaling pathway. Chemokines are the largest subset of chemotactic cytokines, mediating trafficking of different immune cell subsets into the tumor microenvironment and lymphoid tissue development [41]. In addition, chemokines can directly regulate the biological functions of non-immune cells in the tumor microenvironment, including tumor cells and stromal cells. Chemokines and their receptor interactions participate in the tumorigenesis, progression, angiogenesis, and metastasis of various cancer types, including GBM [42]. Some chemokines can be potential diagnostic biomarkers and therapeutic targets for GBM [43]. MerTK is the main receptor of efferocytosis, and it has been proved to be overexpressed in GBM [13]. Additionally, the JAK/STAT signaling pathways, responsive to over 50 cytokines and growth factors, can be amplified by the activation and/or overexpression of MerTK [44]. JAK/STAT signaling pathway, as a central communication node for the immune system, is also associated with glioma cell apoptosis, proliferation, angiogenesis, stem cell maintenance, and immune-suppression [45, 46]. Therefore, it could be speculated that the potential mechanisms of these efferocytosis-related genes were involved in the upregulation of the expression levels of inflammatory mediators, including cytokines and chemokines, through JAK/STAT signaling pathway to promote GBM immune-escape, progression, and invasion.

To understand the relationship between the risk score and GBM microenvironment, the immune/stromal scores were calculated using the ESTIMATE algorithm. It was revealed that the risk score of GBM patients was positively correlated with stromal, ESTIMATE, and immune scores, while negatively correlated with tumor purity. The infiltration levels of 22 immune cells in the two groups were explored using the CIBERSORT algorithm. The results indicated that the infiltration levels of B cell memory, neutrophil, and resting T cell gamma delta were significantly different between the two groups. The expression levels of 35 immune checkpoints, including PD-1, PD-L1, PD-L2, CTLA4, CD276, CD28, and CD27, which have been recently investigated in GBM, were significantly different between the two groups [47]. These findings demonstrated that the prognostic model developed based on the efferocytosis-related genes exhibited a promising performance in distinguishing patients with different immune statuses.

In addition to the direct cytotoxic effects on tumor cells, the subsequent activation of both innate and adaptive anti-tumor immune responses significantly contributes to the therapeutic impact of chemotherapeutic drugs. Through efferocytosis, phagocytes present antigens from apoptotic cells, influencing the immune response either by stimulation or suppression [12]. TMZ serves as the standard first-line chemotherapeutic agent for GBM, and in addition to TMZ, vincristine and carmustine are also preferred agents for recurrent GBM [48]. Utilizing data from the GDSC database, the differences in IC50 values for these three drugs were compared between the two groups. The findings indicated that the low-risk group exhibited greater chemosensitivity to TMZ, vincristine, and carmustine, reflected by lower IC50 values. These results suggest that efferocytosis-related genes may play a role in the chemoresistance of GBM to TMZ, vincristine, and carmustine. Furthermore, tumor cells, adept at remodeling the intricate tumor microenvironment to their advantage, can evade the immune-related benefits of chemotherapy, promoting continued progression and subversion of antitumor immune surveillance [49]. It was hypothesized that differences in the immune microenvironment are partly attributable to the differences in chemotherapy sensitivity between high- and low-risk groups.

We assessed the expression level of 9 PRGs in GBM based on TCGA and GTEx data. The upregulation of PDIA4 in GBM was particularly pronounced when compared to normal tissues. A growing body of evidence underscored the pivotal role of PDIA4 in the endoplasmic reticulum (ER) stress response [24]. In tumor microenvironments, various metabolic abnormalities collaborate to disrupt ER homeostasis in malignant and stromal cells, as well as immunocytes. These conditions induce persistent ER stress, a phenomenon known to modulate several oncogenic traits in cancer cells, including the reprogramming of innate and adaptive immune cells [50]. After confirming the knockdown effect of siRNA-PDIA4 by detecting the expression level of Erp72, the *in vitro* validation of PDIA4’s efficacy in GBM efferocytosis revealed that inhibiting PDIA4 increased PS exposure of GBM cells. However, consistent with bioinformatics analysis, it concurrently decreased efferocytosis and the presence of M2 macrophages in a GBM cells and THP-1 derived macrophage coculture system. Moreover, M2 macrophages secreted factors IL-10 and TGF-β also decreased in PDIA4 knockdown groups. These results reveal that PDIA4 is aberrantly upregulated and expressed in GBM, which leads to more efferocytosis and might endow cancer cells with the ability to resist the endoplasmic reticulum stress (ERS), leading to cancer cell survival in a severe microenvironment [51]. Efferocytosis encompasses multiple phases: the ‘smell phase’ involves phagocytes detecting apoptotic cells and moving towards them; in the ‘eating phase’, phagocytic receptors on phagocytes engage with ligands on apoptotic cells for the specific identification and ingestion process; and the ‘digestion phase’ involves processing the engulfed corpse and its components. While PS exposure is crucial, optimal recognition and ingestion by phagocytes are dictated by a collection of other “eat-me” signals [9]. In addition, efferocytosis induces *naïve* macrophages to adopt an M2 phenotype, which has been exhibited to be a major contributor to poor prognosis by suppressing anti-tumor activity [52]. The specific mechanisms involved in PDIA4-mediated efferocytosis are worthy of further exploration.

Many biomarkers have been reported and are now clinically used in the management of GBM patients. They now play a crucial role in improving diagnostic accuracy, determining prognosis, and predicting treatment responses. In this study, we obtained nine PRGs through screening, which may be used in clinical practice to predict the prognosis of GBM patients. Risk score models constructed based on gene expression may be used in clinical practice to risk stratification of GBM patients and select individuals who are likely to respond to immunotherapy and help medical professionals identify potentially responsive patients and develop effective immunotherapies. At the same time, this nomogram model based on risk score can play an auxiliary role in clinical decision making. To our knowledge, the present study, for the first time, clarified how efferocytosis-related genes could interact with GBM. While this study has several strengths, its limitations are noteworthy. The study limitations were shortly described, firstly the reliance on data mining from a public database with a constrained sample size may induce bias into the prognostic model. Secondly, subsequent experiments are imperative to validate the functional role of core genes and analyze the specific signaling pathways associated with these genes.

In conclusion, a reliable prognostic model comprising efferocytosis-related genes (PDIA4, GNS, OSMR, MXRA8, PDLIM4, STC1, C9orf64, SLC16A13, and GZMB) for GBM patients was developed. Further *in vivo* and *in vitro* studies are required to indicate the precise mechanisms by which these genes participate in GBM progression. Such insights may enhance the therapeutic strategies for GBM.

## MATERIALS AND METHODS

### Data source

Clinical and RNA-seq data were sourced from TCGA database (https://www.ebi.ac.uk/), comprising 167 GBM samples with comprehensive survival details and 5 normal samples. Transcriptomic data of 1034 normal samples were collected from the Genotype-Tissue Expression (GTEx) database. Notably, TCGA-GBM and GTEx-normal datasets were amalgamated to form the training dataset, encompassing 167 GBM samples with complete survival data and 1039 normal samples. Subsequently, the validation dataset was constituted by acquiring transcriptomic data from 137 GBM samples from the Chinese Glioma Genome Atlas (CGGA) database. Following this, efferocytosis-related genes were extracted from previous studies [9, 53].

### Identification and functional enrichment analyses of ERDEGs

Supplementary Table 1 lists the names of R packages and software employed in this study. The batch effect in the training dataset was effectively mitigated using the sva package ComBat seq. Utilizing the DESeq2 R package, DEGs were identified in both normal and GBM samples in the training dataset, adhering to criteria of adj.*P*.value < 0.05 and |log_2_ fold-change (FC)|>1. Visualization of screening results was conducted by generating a volcano map through the ggplot2 R package (ver. 3.3.5), and the expression levels of DEGs were further illustrated using a heatmap generated by the Pheatmap R package (ver. 1.0.12). The exploration of modular genes linked to efferocytosis was carried out through the WGCNA. Initially, outlier samples were filtered out in the training dataset using the Hclust function. Subsequently, the determination of the soft-threshold and adjacency calculation were performed via the pickSoftThreshold function. Identification of modules was undertaken through the dynamic tree cutting approach. Following this, efferocytosis scores for samples in the training dataset were computed using the GSVA R package. The Pearson algorithm was employed to assess the relationship between modules and efferocytosis scores, leading to the selection of the two most relevant modules. The intersection of DEGs between normal and GBM samples and hub genes yielded the identification of ERDEGs. Furthermore, the GO and KEGG pathway enrichment analyses of ERDEGs were carried out using the clusterProfiler R package (ver. 4.0.5).

### Developing a prognostic model for GBM patients

The univariate Cox analysis of DEERGs in TCGA-GBM dataset was employed to obtain ERDEGs associated with GBM patients’ survival. Furthermore, in TCGA-GBM dataset, the LASSO regression analysis was conducted by the glmnet R package to screen out the PRGs of GBM patients and develop a prognostic model. The risk score for each patient was calculated as follows:


$$\sum_{i=1}^{n}\left(coefi\times xi\right)$$

In TCGA-GBM dataset, according to median risk score (−5.23247), GBM patients were categorized into high-risk group (*N* = 83) and low-risk group (*N* = 84). The K-M survival curves were applied to compare GBM patients’ survival between the two groups. Subsequently, the receiver operating characteristic (ROC) curve of the risk score was plotted using the survivalROC R package to assess the function of the risk score. Next, the dependability of the risk score was further assessed in the validation dataset.

In addition, the Wilcoxon rank-sum test was utilized to analyze the risk scores in the training dataset under different clinical characteristics.

### Development of the nomograph model

In TCGA-GBM dataset, it was attempted to explore the independent prognostic value of clinical characteristics and risk score by univariate and multivariate Cox regression analyses. Thereafter, the nomogram model was developed by combining factors of independent prognostic value and the reliability of the nomogram. The model was assessed by the calibration curve.

### GSEA of genes in high- and low-risk groups

In the c2.cp.kegg.v7.5.1.symbols.gmt gene set of TCGA-GBM dataset, the genes from two groups were subjected to GSEA by the clusterProfiler R package.

### The landscape of immune infiltration

In TCGA-GBM dataset, the R estimate package was utilized to calculate and compare tumor purity, stromal, ESTIMATE, and immune scores between high- and low-risk groups. Then, the infiltration levels of 22 immune cells and expression levels of immune checkpoints in the two groups were explored in the training dataset.

### Correlation analysis of risk score with GBM invasion, EMT, and angiogenesis

Invasion-, EMT-, and angiogenesis-associated genes were acquired from Cancer single-cell state atlas (CancerSEA), dbEMT2, and HALLMARK gene sets of the Molecular Signature Database (MSigDB) databases, respectively. In TCGA-GBM dataset, correlations of risk score with invasion-, EMT-, and angiogenesis-associated genes were explored separately by Pearson correlation analysis, and 20 genes with the highest correlation were displayed, separately.

Subsequently, invasion, EMT, and angiogenesis scores were calculated from TCGA-GBM dataset using the single-sample gene set enrichment analysis (ssGSEA). The differences in the above-mentioned three scores were compared between the two groups by the Wilcoxon rank-sum test.

### Drug sensitivity prediction

The IC50 values for TMZ, vincristine, and carmustine in TCGA-GBM dataset were assessed using the pRRophetic algorithm according to the Genomics of Drug Sensitivity in Cancer (GDSC) database. Differences in IC50 values between the two groups were compared by the Wilcoxon rank-sum test.

### Expression levels of PRGs

In the training dataset, differences in the expression levels of the PRGs were compared between GBM and normal samples by the Wilcoxon rank-sum test.

### Cell lines and culture

GBM LN229, A172 cells and human monocytic THP1 cells and were purchased from the Chinese Academy of Sciences Cell Bank (Shanghai, China) and confirmed by Short tandem repeat (STR) analysis. THP1 cells were cultured in a Roswell Park Memorial Institute (RPMI)-1640 medium containing 10% fetal bovine serum (FBS), 0.05 mM 2-mercaptoethanol, 15 mM Hepes, 4.5 g/L glucose, 100 U/ml penicillin, and 100 μg/ml streptomycin (Corning Inc., Corning, NY, USA). LN229 and A172 cells were maintained in a Dulbecco’s modified Eagle’s medium (DMEM) supplemented with 10% FBS, 100 U/ml penicillin, and 100 μg/ml streptomycin (Corning Inc.). Monocytic THP1 cells were differentiated into macrophages by incubation with 40 nM Phorbol 12-myristate 13-acetate (PMA) (MedChem Express, Monmouth Junction, NJ, USA) for 48 h.

### Gene silencing

PDIA4 was knocked down in LN229 and A172 cells using siRNA oligonucleotides (RIBOBIO Biotechnology Co., Ltd., Guangzhou, China). The Lipofectamine 3000 reagent (Invitrogen, Carlsbad, CA, USA) was utilized to transfect cells seeded into six-well plates with 50 nM of oligonucleotides targeting PDIA4, including siPDIA4#1 (5′-GCAAGCGUUCUCCUCCAAUTT-3′), siPDIA4#2 (5′-GCGAGUUUGUCACUGCUUUTT-3′), siPDIA4#3 (5′-CCUGAGAGAAGAUUACAAATT-3′) or the control vector. After 48 h, various transfected cells were validated for PDIA4 knockdown by Western blotting and processed for subsequent experiments.

### Western blotting

Protein was extracted using ice-cold RIPA lysis buffer supplemented with protease and phosphatase inhibitors (Thermo Fisher Scientific, Waltham, MA, USA) and boiled for 10 min at 100°C. Protein lysates were separated by sodium dodecyl sulfate–polyacrylamide gel electrophoresis (SDS-PAGE) and transferred to polyvinylidene difluoride (PVDF) membranes (Merck Millipore, Billerica, MA, USA). After blocking with 5% skim milk at room temperature for 1 h, the membranes were probed with primary antibodies (anti-ERp72 (Cat. No. ab190348, Abcam, UK) and anti-GAPDH (Cat. No. ab190348, Abcam)) at 4°C overnight, followed by incubation with HRP-conjugated anti-rabbit secondary antibody at room temperature for 1.5 h. SuperSignal^™^ West Pico PLUS (Thermo Fisher Scientific) was utilized to enhance the chemiluminescence, and the membranes were visualized by the ChemiDoc Imaging system (Bio-Rad Laboratories Inc., Hercules, CA, USA).

### Flow cytometry

Efferocytosis was assessed in macrophages using apoptotic LN229 and A172 cells line and quantified by flow cytometry. Apoptosis of LN229 and A172 cells was induced by 15 μM TMZ (MedChem Express) for 24 h. The exposure of PS on apoptotic cell surface was detected using the Annexin V APC ready flow kit (Invitrogen). Apoptotic LN229 and A172 cells were labeled with CSFE (Invitrogen) and cocultured with macrophages in above-mentioned medium for THP-1 at the ratio of 1:1 for 24 h. Cocultured medium was collected for cytokine analysis, and cocultured cells were subsequently washed with 0.2% BSA-PBS. Cell pellets were incubated with APC labeled CD11b primary antibody (BioLegend, San Diego, CA, USA) in the dark for 40 min, followed by thrice rinsing with 0.2% BSA-PBS. The efferocytosis activities were measured by a flow cytometer (Becton Dickinson, Franklin Lakes, NJ, USA) and analyzed by FlowJo software (Three Star, Inc., Ashland, OR, USA).

Cell surface expression level of CD206 was quantified by flow cytometry. THP-1-derived macrophages were seeded into a 0.4-μm Transwell insert (Merck Millipore) with a RPMI-1640 medium and then cocultured with transfected LN229 and A172 cells for 48 h. Macrophages were collected and labeled with PE-conjugated anti-CD206 antibody (BioLegend). After 45 min of incubation, CD206 expression level was determined by a flow cytometer.

### Chemokine analysis

The cocultured medium from efferocytosis assays underwent analysis for detecting the levels of IL-10 and TGF-β1 using the human IL-10 ELISA kit (Abcam) and TGF-β1 ELISA kit (Abcam), respectively, following the manufacturer’s protocols on Infinite 200Pro (Tecan, Männedorf, Switzerland). All experiments were conducted in triplicate and repeated three times.

### Statistical analysis

All statistical analyses of bioinformatics analysis section in this study were performed in R software (version 4.1.0). The Wilcoxon rank-sum test was utilized to analyze the risk scores under different clinical characteristics, the differences in three scores, the differences in IC50 values, differences in the expression levels of the PRGs were compared between GBM and normal samples. The Pearson algorithm was employed to assess the relationship between modules and efferocytosis scores, correlations of risk score with invasion-, EMT-, and angiogenesis-associated genes. LASSO-Cox regression was used for efferocytosis-related genes selection. The Kaplan-Meier method was used to compare the survival rate between the low- and high-risk groups. Univariate and multivariate Cox regression analyses were used to assess the independent prognostic variables.

For cell experiments, data were presented as mean ± SD and analyzed by two-tailed unpaired *t* test using Graphpad Prism 7.0 (La Jolla, CA, USA).

### Data availability

The datasets used and analyzed in the current study are available from the GDSC database (http://www.cancerrxgene.org/), TCGA database (https://www.ebi.ac.uk/), GTEx database (http://commonfund.nih.gov/GTEx/), CGGA database (http://www.cgga.org.cn/), MSigDB database (http://software.broadinstitute.org/gsea/msigdb/index.jsp).

## Supplementary Materials

Supplementary Table 1
