# Supplementary material for: Development of a prognostic model for glioblastoma multiforme based on the expression levels of efferocytosis-related genes
Source: Aging (Albany NY). 2023 Dec 29;15(24):15578–98. doi: 10.18632/aging.205422 (PMC10781462; doi:10.18632/aging.205422)
Supplement: Supplementary Table 1 [file aging-15-205422-s001.pdf]

SUPPLEMENTARY TABLE

Supplementary Table 1. The list of R packages and software used in this study.

R package/software	Version	Link
ClusterProfiler	4.0.5	https://git.bioconductor.org/packages/clusterProfiler
DESeq2	1.34.0	https://github.com/mikelove/DESeq2
ggplot2	3.3.5	https://github.com/tidyverse/ggplot2
pheatmap	1.0.12	https://github.com/raivokolde/pheatmap
ggvenn	0.1.9	https://github.com/yanlinlin82/ggvenn
survival	3.2–13	https://github.com/therneau/survival
survminer	0.4.9	https://github.com/kassambara/survminer
forestplot	2.0.1	https://github.com/gforge/forestplot
GSVA	1.42.0	https://github.com/rcastelo/GSVA
glmnet	4.1–2	https://glmnet.stanford.edu/
pRRophetic	0.5	https://osf.io/5xvsg/wiki/home/
rms	6.2–0	https://hbiostat.org/R/rms/
estimate	1.0.13	https://r-forge.r-project.org/projects/estimate/
